# Information and order of information effects on consumers’ acceptance and valuation for genetically modified edamame soybean

**DOI:** 10.1371/journal.pone.0206300

**Published:** 2018-10-24

**Authors:** Ji Yong Lee, Michael P. Popp, Elijah J. Wolfe, Rodolfo M. Nayga, Jennie S. Popp, Pengyin Chen, Han-Seok Seo

**Affiliations:** 1 Department of Agricultural Economics and Agribusiness, University of Arkansas, Fayetteville, Arkansas, United States of America; 2 Division of Plant Science, University of Missouri – Delta Center, Portageville, Missouri, United States of America; 3 Department of Food Science, University of Arkansas, Fayetteville, Arkansas, United States of America; University of Tsukuba, JAPAN

## Abstract

This study examines two different strategies with respect to managing the order in which information about genetically modified (GM) technology would reach and impact consumers of edamame, often referred to as the “vegetable soybean”. Edamame are soybeans harvested while the beans are young and soft. We capture consumers’ willingness to pay (WTP) for unlabeled edamame, non-GM edamame, and GM edamame using a non-hypothetical random *n*^*th*^ price auction. We elicit consumers’ valuation for each edamame product before and after introducing information, and test two strategies where the order of providing positive and negative information is reversed. The results suggest that negative information affects WTP to a much greater extent than positive information. Hence a strategy to proactively deal with eventual negative press about GM technology did not lead to a different result than a strategy that would react to or attempt to thwart negative information with positive information at a later date. These findings suggest that it would be difficult to introduce new GM edamame as edible products in the market as marginally negative preconceptions about GM at the time of the experiment were easier to reinforce with negative information than to combat with positive information about GM.

## Introduction

Edamame, also known as vegetable soybean, is soybean harvested while the beans are young and soft. Although the United States (US) is the world’s leading producer of soybean (*Glycine Max* L.), it lags behind a number of Asian countries in the production of edamame. Nonetheless, edamame production has been increasing in the US given growing consumer interest. While Edamame is not tracked as a separate vegetable crop by United States Department of Agriculture (USDA) National Agricultural Statistics Service to date, sales of frozen edamame was reported to have increased 40% from 2003 to 2007 in the US [1]. Although approximately 94% of total soybean production is planted with genetically modified varieties in the US [2], edamame is currently being commercially processed using exclusively non-genetically modified (GM) edamame cultivars. While soybean for feed and food ingredients are extensively bred using GM technology, future breeding efforts toward developing GM edamame, that are currently not active, to lower cost of production, increase yield, or enhance other desirable product characteristics, could be thwarted by consumer resistance toward GM vegetables.

Also, with the debate intensifying over mandatory genetically modified (GM) labeling, soybean growers, breeders, processors, and marketers need information on how consumers react to different types of messages about GM. Information about new and existing GM technologies can influence consumer perceptions and their purchasing behaviors. Some previous studies have examined the effect of information about GM on consumers’ acceptance of GM foods [3–7]. For example, Lusk et al. [3] examined the effect of information about potential benefits of GM (i.e., environmental benefit, health benefit, world benefit) on consumer preference for GM foods (i.e., chocolate chip cookie containing GM ingredients) in both the US and European countries. They found that positive information about GM has a positive impact on the acceptance of GM, but these effects can differ across locations and cultures. Moreover, they found that certain types of information have a greater impact on consumer valuation. Rousu et al. [4] investigated the effect of both positive and negative information about GM from different group perspectives (i.e., environmental group, biotech industry, and third-party group) on consumers’ valuation for GM foods (i.e., vegetable oil, tortilla chips, and Russet potatoes) and found that consumers with positive information increase their valuation for GM food relative to non-GM food while consumers with negative information decrease their valuation for GM food. In addition, they found that the effect of negative information outweighs that of positive information, but that verifiable third-party information reduces the effect of negative information. Corrigan et al. [5] investigated consumer preference for GM golden rice and examined the effects of positive and negative information about GM (i.e., food safety, human impact, socio-economic impact, and environmental impact) from two different group perspectives (i.e., Golden rice board, Greenpeace) on consumer valuation for GM golden rice. They also found that consumers place a greater weight on negative information compared to positive information. Thorne et al. [6] examined Irish consumers’ acceptance of GM late blight resistant potatoes and the effect of information about potential health and economic benefits of using GM on consumers’ purchasing decisions. They found that consumers generally prefer conventional potatoes to GM potatoes, but after receiving favorable information about GM, they significantly increased their valuations for GM potatoes.

Given consumers’ sensitive response to information about GM technology and the stronger effects of negative information on product choices, the question of how to disseminate different types of information is important, especially when introducing a new GM product in the market. Previous studies have provided evidence that people have a tendency to place more weight on more recently acquired information in their decision making since it remains in short-term memory compared to the older information [8–10]. The findings from previous studies suggest that the order of disseminating information (e.g., positive information first and negative information later or negative information first and positive information later) may have different impacts on consumers’ acceptance/choice of new product. Understanding the effects of such information dissemination is important since stakeholders/marketers are interested in whether or not to introduce their products and how they should effectively advertise or promote their product under a market environment with imperfect information.

This study seeks to determine whether the order of disseminating different types of information on GM technology affects consumer choice and acceptance of a novel GM product. Markets for controversial food attributes or processes such as GM have been characterized as having disparate information. Given the conflicting market environment about GM foods, the question of how to effectively reconcile divergent views is important. A Proactive strategy would be to provide educational information emphasizing mainly positive rationale for choosing a technology or a product early on as a means to manage potential backlash about controversial issues later on by being transparent up front. A Reactive strategy would be to react to negative information in the market place by highlighting positive aspects of a technology after negative aspects have been released. Hence, the first objective of this study is to investigate the effects of both positive and negative information about GM technology on consumers’ valuation for GM edamame which does not exist to date. Second, this study identifies consumers’ valuation behavior when the order in which information is provided is changed (i.e., the positive information is given first, followed by the negative information, or, the negative information is given first, followed by the positive information).

To investigate the effects of different types of information on consumer acceptance of GM edamame, we used a non-hypothetical experimental auction (i.e., the random nth price auction using a full bidding approach) and elicited consumers’ willingness to pay (WTP) before and after introducing information. In addition, we test Proactive vs. Reactive strategies where the order of providing positive and negative information about GM technology was reversed.

## Materials and methods

### Experimental procedure

The experiment was reviewed and approved by the Institutional Review Board (IRB) at The University of Arkansas in the U.S. The subjects voluntarily participated in the study and they were free to withdraw from the study at any point with no negative repercussions. As detailed in Wolfe [11], participants were randomly recruited from a consumer database at a large state university. The only requirement for participants was that they had no soy allergies. The age range of the recruited participants was between 25 and 54. We conducted a sensory test prior to the experimental auction to elicit participants’ sensory responses and to make the experiment more realistic. The results of the sensory tests for the GM and non-GM soybeans harvested at the edamame stage (hereafter referred to as GM and non-GM edamame) are not reported here since they had nearly identical sensory evaluations. Respondents then participated in an experimental auction and filled out a short questionnaire that contained questions related to GM foods and their demographics. Our sample consisted of 117 participants. The experiment included two treatments that differed in the order of providing information about GM: a Proactive strategy where positive information was provided first and followed by negative information, and a Reactive strategy where negative information was introduced first and followed by positive information. Before the experiment began, instructions were clearly explained and participants were required to sign an informed written consent form. Once the experiment was completed, each participant was given a $25 gift card as payment for participating in the study; i.e., to cover their opportunity cost associated with spending time on the experiment.

The participants were asked to evaluate three edamame products: GM labeled edamame, non-GM labeled edamame, and an unlabeled edamame. The random n^th^ price auction was used to identify consumers’ valuation for each of these three products. This auction mechanism is incentive compatible and widely used in valuation studies [12–14]. In a random n^th^ price auction, each participant places a confidential bid on the item(s) being auctioned. The bids are then ranked from highest to lowest, and a random number (n) is selected by the experimenter from two to the total number of bidders in the auction. Subsequently, the n^th^ highest bid becomes the market price that anyone who bids above it has to pay. Therefore, there are (n-1) individuals who can buy the product auctioned for (i.e., the (n-1) highest bidders) and each of them will pay the nth highest bid as the price for the product.

The experimental procedures were read aloud to the participants in each session. Using a framework similar to Wszelaki et al.’s study [15], the sensory test was conducted at first to allow each participant to taste the GM and non-GM edamame in a sensory testing booth. Following the sensory test, a hypothetical candy bar auction and quiz were used as teaching instruments to make sure that all participants clearly understood the auction mechanism and procedures before the actual random n^th^ price auction occurred for the GM, non-GM, and unlabeled edamame products. The candy bar practice auction was of the same format as the eventual edamame auction, except for the actual payment for and distribution of the candy bars.

As previously mentioned and as depicted in Table 1, the participants were randomly assigned to two treatments (i.e., proactive and reactive treatments). The auction used three bidding rounds with simultaneous bids on the three products of interest: GM labeled edamame, non-GM labeled edamame, and unlabeled edamame. For the Proactive treatment, no information was given in the first bidding round, i.e., participants were only shown the three products with no information provided, and then positive information about GM technology was given in the second bidding round, followed by negative information in the last bidding round. For the Reactive treatment, no information was provided in the first bidding round, followed by negative information in the second bidding round, and positive information in the third bidding round. We provided subjects ample time to read and process the information before asking them to submit their bids for the products. The positive and negative information given to subjects are exhibited in Table 1. The participants were informed that one of the three products would be randomly chosen as the binding product after the three auction rounds. Similarly, they were also informed that one of the three rounds would be randomly selected as the binding round. Hence, the number of winners in the auction was the top (n-1) bidders of the binding product in the binding round. Each of them got the binding product and paid the nth highest bid.

**Table 1:** Information treatments.

*Treatment 1 – Proactive*
• Round 1 – No information
• Round 2 – Positive information
• Round 3 – Negative information
*Treatment 2 – Reactive*
• Round 1 – No information
• Round 2 – Negative information
• Round 3 – Positive information
*Positive Information +*
Genetically engineered soybean food products are cheaper to produce as more effective herbicides can be sprayed over a larger window of time. This leads to higher yields and greater producer flexibility in managing production. It also lessens the amount of resources needed per amount of edible food as fewer inputs are needed. This helps lower the carbon footprint of edamame. (Source: Nalley et al. [16])
*Negative Information –*
Today’s use of genetically engineered seed allows producers to apply herbicides to control weeds and/or pests that would normally also kill soybeans. An unintended side effect of this technology has been the growing weed/pests tolerance to these herbicides/pesticides as well. As a result, farmers now use more herbicide/pesticides and also pay higher prices for biotech seed causing their profit margins to decline. (Source: Norsworthy et al. [17] and Riar et al. [18])

Demographic questions on gender, age, education level, presence of children in the household, knowledge of and opinion about GM foods, household size, and frequency of quarterly edamame consumption were collected after the auction using a questionnaire (S1 Appendix. Experimental Instructions and Survey Questionnaire).

### Preparation of edamame samples

Subjects were informed that GM and non-GM soybeans with cultivars exhibiting similar pod and seed size at the end of the pod filling stage were grown near a land grant university’s sensory research center and were harvested at the optimum edamame stage. Once harvested, the edamame were (1) blanched at 100°C for 90 seconds to sufficiently inactivate lipoxygenase activity before packaging to keep the edamame pods’ desirable green color and textural attributes (Mozzoni et al. [19]); (2) packaged in clear, 8 oz. (237 mL) bags containing approximately 40–50 pods, and (3) vacuum sealed. The packages were then frozen and labeled as GM, non-GM, and unlabeled, with the unlabeled product being randomly filled with GM or non-GM edamame.

### Statistical analysis

To test the effects of both positive and negative information on consumer valuation for GM, non-GM, and unlabeled edamame, unconditional t-tests were used to quantify differences in responses. We also utilized the same unconditional t-tests to compare the effects of the order of providing information about GM on consumers’ value changes for GM edamame. We then used two-sample proportion tests to examine whether information order had an impact on the incidence of product choice changes. Since consumer values and changes in values can be influenced by their heterogeneous characteristics, we used conditional regression models such as Tobit and Probit models to further examine impacts of information and the order of providing information when demographic, behavioral and attitudinal variables were included. We used Stata 15.0 by StataCorp to conduct both unconditional and conditional tests.

## Experimental results

Table 2 compares the characteristics of the sample of participants randomly assigned to the treatments. The following t-statistics and p-values show the results of mean equality tests between the two treatments. About 77 percent and 70 percent of participants were female in the Proactive and Reactive treatments, respectively (t-statistic: 0.88, p-value: 0.38). The average ages of participants were about 37 years in the Proactive group and about 39 years in the Reactive group (t-statistic: 0.13, p-value: 0.26). About half of the participants had bought edamame to prepare meals for their household in the past three months (t-statistic: 0.44, p-value: 0.65). Participants assessed their knowledge level as somewhat informed about GM foods (t-statistic: 1.22, p-value: 0.22), and they expressed a near neutral attitude with slight negative sentiment toward GM foods (t-statistic: 0.78, p-value: 0.44). While income level across treatments was slightly different, the mean difference was not statistically significant (t-statistic: 1.42, p-value: 0.16). Overall, the sample characteristics between the two treatments are similar, suggesting that the randomization procedure successfully balanced the observed characteristics across the information order treatments.

**Table 2:** Comparison of participants’ characteristics across information order treatments^a^.

		Proactive		Reactive	
Variable	Categories	Mean	Std. Dev.	Mean	Std. Dev.
Gender	1: Female, 0: Male	0.77	0.42	0.70	0.46
Age	Years	36.51	11.89	38.63	8.01
Children	1: Yes, 0: No	0.42	0.49	0.55	0.50
BFrequency^b^	1: At least one time in the past three months, 0: Never	0.51	0.50	0.55	0.50
Knowledge^c^	1: Not at all informed to 5: Extremely well informed	2.89	0.84	2.70	0.88
Attitude^d^	1: In favor of GM to 4: Against GM	2.54	0.48	2.61	0.48
Education	Less than Bachelor’s degree	47.3%		48.3%	
	Bachelor’s degree	23.6%		26.7%	
	Master’s degree or higher	29.1%		25.0%	
Income	Less than $2,999 per month	45.4%		33.3%	
	$3,000–$5,999	40.0%		43.3%	
	More than $6,000	14.5%		23.3%	

^a^ See Table 1 for definition of information order treatments.

^b^ Frequency of purchasing edamame to prepare meals in the past three months.

^c^ Respondents’ self-reported level of knowledge about GM foods.

^d^ See S2 Appendix. Question Used to Form Attitude Variable towards GM Food.

### Overview of consumers’ values and information effect

Participants’ bids are summarized in Table 3. Focusing first on valuation for GM edamame, it is clear that negative information on GM has a greater impact on consumer valuation for GM edamame compared to positive information in both treatments. However, these information effects are not statistically significant (*positive information*: t-statistic: 0.11, p-value: 0.91 for Proactive strategy; t-statistic: 0.19, p-value: 0.85 for Reactive strategy; *negative information*: t-statistic: 0.99, p-value: 0.32 for Proactive strategy; t-statistic: 1.44, p-value: 0.15 for Reactive strategy).

**Table 3:** Consumer valuation for 8oz. Packages of GM, Non-GM, and Unlabeled Edamame by Information Order Treatment^a^.

	Proactive: None fb + fb -			Reactive: None fb – fb +		
	GM Edamame					
	Mean	Median	Std. Dev.	Mean	Median	Std. Dev.
Round 1	$1.00	$0.75	$1.11	$1.08	$1.00	$1.00
Round 2	$0.98	$0.75	$1.15	$0.82	$0.50	$0.93
Round 3	$0.78	$0.25	$0.93	$0.85	$0.50	$0.97
	Non-GM Edamame					
	Mean	Median	Std. Dev.	Mean	Median	Std. Dev.
Round 1	$1.28	$1.00	$1.03	$1.52	$1.50	$1.19
Round 2	$1.23	$1.00	$1.00	$1.54	$1.40	$1.18
Round 3	$1.37	$1.00	$1.17	$1.51	$1.28	$1.17
	Unlabeled Edamame					
	Mean	Median	Std. Dev.	Mean	Median	Std. Dev.
Round 1	$0.89	$0.75	$0.94	$1.12	$1.00	$0.99
Round 2	$0.87	$0.50	$0.94	$1.00	$0.88	$0.98
Round 3	$0.94	$1.00	$1.01	$0.98	$0.90	$0.99

^a^ Information order treatment was no information (None) for first round bids, followed by (fb) positive (+) information prior to 2^nd^ round bids fb negative(-) information prior to 3^rd^ round bidding in the first treatment. The second treatment reversed the information treatment prior to 2^nd^ and 3^rd^ round bidding as indicated (Table 1).

The bid results also show that positive information on GM reduces consumer valuation for non-GM edamame while negative information on GM leads to an increase in value for non-GM edamame as expected. However, the changes in values are not statistically significant in both cases. For unlabeled GM, a similar trend of value change is observed as with non-GM for the Proactive strategy. However, negative information about GM reduced the value for unlabeled GM while positive information lead to an increase in the value for the Reactive strategy. Again, the changes in values are not statistically significant in both cases.

Next, we examine whether the order of providing information about GM has a differential impact on consumers’ total value changes for GM edamame. Table 4 reports the effects of order of providing information on value changes. Results show that value changes (i.e., bid differences) between round 3 (R3) and round 1 (R1) are similar between the two information order treatments. According to the t-test, we fail to reject the equality of mean value change between the two treatments (t-statistic: -0.03, p-value: 0.97), indicating that consumers symmetrically respond to information regardless of the order in which they were provided.

**Table 4:** Effect of order of providing information on valuation change for GM edamame.

Information Treatment^a^	Information Effect	Value Change^b^	Mean	Std. Dev.
Proactive: None fb + fb - (N = 57)	Positive	R2 –R1	-0.02	0.33
	Negative	R3 –R2	-0.20	0.79
	Combined	R3 –R1	-0.22	0.78
Reactive: None fb - fb + (N = 60)	Negative	R2 –R1	-0.26	0.62
	Positive	R3 –R2	0.03	0.36
	Combined	R3 –R1	-0.22	0.72

^a^ Information order treatment was no information (None) for first round bids, followed by (fb) positive (+) information prior to 2^nd^ round bids fb negative(-) information prior to 3^rd^ round bidding in the first treatment. The second treatment reversed the information treatment prior to 2^nd^ and 3^rd^ round bidding as indicated (Table 1).

^b^ Bid differences between rounds. R1, R2, and R3 represent Round 1, Round 2, and Round 3.

**Fig 1:**
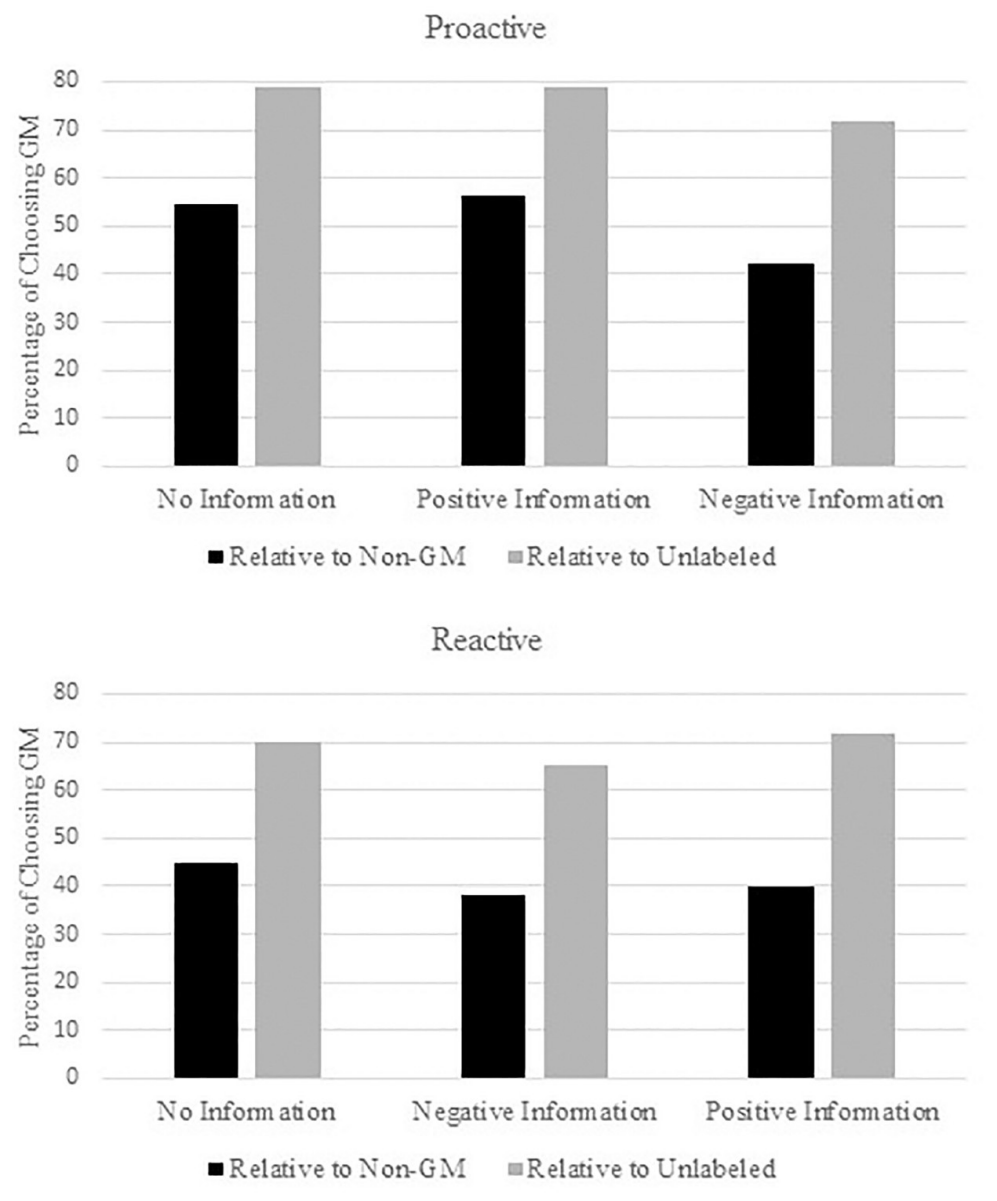
Effect of information on relative preference of GM to non-GM and unlabeled edamame by information strategy.

We also investigated whether information order about GM had an impact on the incidence of choice changes. We define the incidence of choice change if consumers changed their preference from GM edamame to non-GM or unlabeled edamame after receiving information. Fig 1 exhibits the percentage of choosing GM edamame relative to each of non-GM and unlabeled edamame on the basis of WTP. We define the percentage of choosing GM edamame when consumers’ values for GM edamame is the same or higher than each of non-GM or unlabeled edamame. From the figure, it is clear that consumers relatively more choose GM edamame compared to unlabeled edamame regardless of information treatment. This may due to the fact that unlabeled is considered unknown and therefore a risk to some consumers. Consumers generally prefer non-GM edamame to GM edamame, especially with negative information about GM technology.

Focusing on the information effect in the Proactive strategy, positive information on GM barely raised the percentage of choosing GM edamame relative to non-GM edamame and did not affect choice in comparison to the unlabeled edamame while negative information on GM lead to a decrease in the percentage of choosing GM edamame as expected. The two-sample proportion tests however fail to reject the null hypothesis of equal proportions between the information rounds (*positive information*: z-statistic: -0.19, p-value: 0.85 in the choice of GM relative to Non-GM; z-statistic: 0.00, p-value: 1.00 in the choice of GM relative to unlabeled edamame; *negative information*: z-statistic: 1.49, p-value: 0.13 in the choice of GM relative to Non-GM; z-statistic: 0.87, p-value: 0.38 in the choice of GM relative to unlabeled edamame). For the Reactive strategy, the percentage of choosing GM edamame decreases after receiving negative information, and it recovers after receiving positive information about GM. Again, the proportion tests fail to reject the null hypothesis of equal proportions (*positive information*: z-statistic: -0.18, p-value: 0.85 in the choice of GM relative to Non-GM; z-statistic: -0.78, p-value: 0.43 in the choice of GM relative to unlabeled edamame; *negative information*: z-statistic: 0.74, p-value: 0.45 in the choice of GM relative to Non-GM; z-statistic: 0.58, p-value: 0.55 in the choice of GM relative to unlabeled edamame).

### Conditional regression analysis of information effect

The descriptive statistics and unconditional tests do not completely reveal the impacts of different types of information and the order of providing them. Therefore, we examined our objectives at the individual levels by estimating conditional regression models. To compare the effects of positive and negative information about GM on consumer valuation for GM edamame, we used the Tobit specification since individuals’ bids for GM edamame were censored at zero given our experimental setting (i.e., subjects can bid zero for the products). Specifically, in our bid data for GM edamame, about 36 percent of all bids were zero in the first bidding round, and about 44 percent of all bids were zero in the second and third bidding rounds. The model was estimated using the bids for GM edamame using all bidding rounds. To take into account the panel nature of our data, we used the random effects Tobit regression models.

$${GM}_{iR}=\alpha +\beta_{1}{PDum}_{i}+\beta_{2}{NDum}_{i}+\beta_{3}X_{i}+u_{i}+\varepsilon_{iR}$$

where *GM*_*iR*_ is an individual’s bid for GM edamame in bidding round R; *PDum*_*i*_ is a binary variable = 1 if an individual receives positive information and 0 otherwise; *NDum*_*i*_ is a binary variable = 1 if an individual receives negative information and 0 otherwise; *X*_*i*_ denotes a vector of control variables that include general socio-demographic factors such as gender, age, education level, income level, and presence of children in the household; other individual characteristics such as frequency of buying edamame, level of knowledge about GM, and attitude toward GM; *u*_*i*_ is random effects which control for unobservable individual characteristics; and *ε*_*iR*_ is i.i.d. component.

Table 5 shows estimation results for each treatment. Focusing initially on the comparison of the effects of positive and negative information when first applied in the second bidding round (*Positive 1*^*st*^ and *Negative 1*^*st*^) in the rightmost column labeled ‘Pooled’ where data from both treatments are used, positive information had essentially no effect to WTP for GM edamame whereas the negative information significantly lowered WTP. In addition, the effect of negative information is significantly greater in absolute terms (Wald test of coefficient estimates of *Positive 1*^*st*^ and *Negative 1*^*st*^ in the pooled model (*χ*^2^: 3.79, p-value: 0.05)). This finding is consistent with other previous studies on the effect of information in product valuation—for example, Corrigan et al. [5] found that consumers place more weight on negative information than positive information when they value GM golden rice.

**Table 5:** Effect of information strategy on valuation of GM edamame.

	Proactive	Reactive	Pooled
	Coefficient (Std. Err.)	Coefficient (Std. Err.)	Coefficient (Std. Err.)
*Positive 1*^*st*^	-0.07 (0.14)	--	-0.06 (0.13)
*Negative 2*^*nd*^	-0.40 (0.14)***	--	-0.39 (0.14)***
*Negative 1*^*st*^	--	-0.42 (0.12)***	-0.42 (0.12)***
*Positive 2*^*nd*^	--	-0.35 (0.12)***	-0.35 (0.12)***
*Gender*	-0.94 (0.59)	0.07 (0.43)	0.01 (0.35)
*Age*	-0.04 (0.02)*	0.02 (0.02)	-0.01 (0.01)
*Education*	-0.06 (0.21)	-0.06 (0.15)	-0.07 (0.12)
*Income*	-0.14 (0.13)	0.01 (0.08)	-0.04 (0.07)
*Children*	0.88 (0.51)*	-0.62 (0.37)*	-0.04 (0.32)
*BFrequency*	-0.08 (0.39)	0.43 (0.29)	0.37 (0.25)
*Knowledge*	-0.39 (0.28)	0.17 (0.21)	-0.10 (0.17)
*Attitude*	-1.01 (0.51)**	-0.78 (0.39)**	-0.75 (0.33)**
*Treatment 2*	--	--	0.30 (0.32)
*Intercept*	6.86 (2.38)***	1.10 (1.58)	3.36 (1.44)**
N. of Obs.	171	180	351
Log likelihood	-167.72	-180.52	-357.87

Note:

*, **, and *** denote significance levels at 10%, 5%, and 1%, respectively. *Positive 1*^*st*^ and *Negative 1*^*st*^ represent the information provided first in each treatment. *Positive 2*^*nd*^ and *Negative 2* ^*nd*^ denote the information provided later in each treatment. See also Tables 1 and 2 for information strategy and variable descriptions.

For the Proactive information strategy, positive information had no significant impact on consumer valuation for GM edamame while negative information significantly reduced consumer valuation with the effect of positive information accounted for. This evidence is in line with Fig 1 and is also evident in the coefficient estimates for the pooled model in the right column of Table 5. This is consistent with the theory that an individual places more weight on the most recently acquired information in their decision making.

Focusing on the Reactive information strategy in the second column of Table 5, positive information (*Positive 2*^*nd*^), after the effects of first receiving negative information, did lead to a rebound in WTP from -0.42 to -0.35 suggesting the Reactive strategy may be superior to the Proactive strategy given the smaller overall negative impact in the reactive model. A Wald test of coefficient estimates of *Positive 2*^*nd*^ to *Negative 1*^*st*^ in the Reactive model (*χ*^2^: 0.33, p-value: 0.56) suggests that this change in WTP was not statistically significant. This result suggests that negative information is difficult to reverse.

Finally, a comparison of the *Positive 2*^*nd*^ to the *Negative 2*^*nd*^ coefficients in the pooled model revealed no statistical significance (Wald test of coefficient estimates of *Positive 2*^*nd*^ and *Negative 2*^*nd*^ (*χ*^2^: 0.05, p-value: 0.82)). Hence, it does not matter whether a proactive or reactive strategy is pursued. It is commonly known that negative information is weighted more heavily than positive information in people’s decision making (referred to as *negativity bias*) [20–22]. Given this negativity bias, our results suggest that the order of disseminating information would not be matter, at least in our case; people tend to put more weight on negative information than positive information.

Further regression analysis on the order of providing information examines how the total value changes for GM edamame after all information is used by the respondents over time. In the analysis, we treat the Reactive information strategy group as the baseline and include a binary variable for the Proactive group to determine if the coefficient on the latter is positive and statistically significant.

$${CValue}_{i}=\alpha +\beta_{1}{DPosNeg}_{i}+\beta_{2}X_{i}+\epsilon_{i}$$

where *CValue*_*i*_ is an individual’s value change between the third and first rounds of bidding for GM edamame; *α* captures the baseline Reactive information strategy effect of all information; *DPosNeg*_*i*_ is the dummy for the proactive group who received positive information first and negative information later; *X*_*i*_ is the vector of control variables explained in the previous model; *ϵ*_*i*_ is an i.i.d. error term.

Table 6 provides the regression results. The results confirm our observation that individuals respond to positive and negative information the same regardless of the order in which the information is provided. The result is consistent with the above analyses in that positive information essentially had no significant impact on WTP whereas negative information had the expected effect on WTP and was overpowering relative to the positive information.

**Table 6:** Effect of order of providing information on total value change.

	Coefficient	Standard Error
*DPosNeg*	-0.05	0.12
*Gender*	-0.09	0.14
*Age*	0.02	0.01
*Education*	0.04	0.05
*Income*	-0.05*	0.03
*Children*	-0.35***	0.11
*BFrequency*	-0.03	0.13
*Knowledge*	0.07	0.05
*Attitude*	-0.14	0.09
*Intercept*	-0.39	0.59
N. of Obs.	117	
R-Squared	0.18	

Note:

* and *** denote significance levels at 10% and 1%, respectively.

Finally, we investigate whether information impacts the incidence of choosing GM edamame relative to non-GM and unlabeled edamame using Probit models. Results are shown in Table 7. The dependent variable in models (1) and (3) is a binary value = 1 if individuals’ values for GM edamame are the same as or greater than values for non-GM edamame and 0 otherwise. The dependent variable in models (2) and (4) is a binary variable = 1 if individuals’ values for GM edamame are the same as or greater than values for unlabeled edamame and 0 otherwise. The results again confirm that negative information about GM lowers the probability of choosing GM edamame relative to non-GM and unlabeled edamame; however, it is only statistically significant in model (1). Positive information about GM does not significantly increase the probability of choosing GM edamame in all models. Hence a Proactive strategy will not protect against potential negative information that may well follow announcement of positive information about GM technology.

**Table 7:** Effects of information on incidence of choosing GM edamame.

	Proactive		Reactive	
	(1) Probability of Choosing GM relative to Non-GM	(2) Probability of Choosing GM relative to Unlabeled	(3) Probability of Choosing GM relative to Non-GM	(4) Probability of Choosing GM relative to Unlabeled
*Positive 1*^*st*^	0.16 (0.41)	-0.01 (0.44)	--	--
*Negative 2*^*nd*^	-1.00 (0.44)**	-0.64 (0.44)	--	--
*Negative 1*^*st*^	--	--	-0.42 (0.34)	-0.28 (0.34)
*Positive 2*^*nd*^	--	--	-0.30 (0.34)	0.13 (0.35)
*Gender*	2.00 (1.17)*	1.71 (1.04)*	1.36 (0.76)*	0.47 (0.65)
*Age*	-0.02 (0.03)	-0.03 (0.03)	0.03 (0.04)	-0.02 (0.03)
*Education*	-0.79 (0.41)*	-0.81 (0.39)**	0.02 (0.26)	0.10 (0.24)
*Income*	0.09 (0.23)	0.24 (0.23)	-0.03 (0.15)	-0.05 (0.13)
*Children*	0.07 (0.95)	-0.52 (0.88)	0.25 (0.63)	-0.69 (0.59)
*BFrequency*	-0.13 (0.83)	-0.81 (0.78)	-0.89 (0.69)	-0.66 (0.63)
*Knowledge*	0.09 (0.51)	-0.41 (0.49)	-0.71 (0.37)*	-1.16 (0.39)***
*Attitude*	-4.21 (1.35)***	-4.03 (1.38)***	-1.37 (0.71)*	0.03 (0.57)
*Intercept*	12.85 (4.83)***	16.21 (5.48)***	3.32 (2.85)	5.10 (2.49)**
N. of Obs.	171	171	180	180
Log likelihood	-70.93	-56.71	-86.14	-80.74

Note:

*, **, and *** denote significance levels at 10%, 5%, and 1%, respectively. *Positive 1*^*st*^ and *Negative 1*^*st*^ represent the information provided first in each treatment. *Positive 2*^*nd*^ and *Negative 2*^*nd*^ denote the information provided later in each treatment. See also Tables 1 and 2 for information strategy and variable descriptions.

## Conclusions and discussion

In this study, we conducted experimental auctions to identify the effects of positive vs negative information about GM technology on consumers’ valuation for a novel GM product. Given the theory that people place greater weight on more recently acquired information than older information in their decision-making process, we specifically investigated whether the order of providing information matters in their valuation for the product.

The results showed that consumers place more weight on negative information than positive information about GM. Specifically, positive information about GM did not significantly increase consumer valuation of GM edamame while negative information significantly reduced their valuation. More importantly, the effect of negative information persisted even after providing positive information. The tendency of the persistent negative information effect even after the provision of positive information has implications for the design of information strategies. One of the implications from the previous studies is that more information about GM technology should be available and easily accessible to consumers. Our finding suggests that this information strategy may not be enough to promote/advertise new GM foods given the greater effect of negative information. According to Lusk et al. [3, 23, 24], consumers’ acceptance of GM foods is heterogeneous across the benefits of the GM technology. Specifically, consumers have more acceptance of GM technology when application of GM provides tangible benefits to them. For example, consumers’ acceptance of GM foods increases when the application of GM provides nutritional benefits [3, 23, 25], environmental benefits [3, 26, 27], lowering pesticide residues [23, 26], and food security in developing countries [3, 28]. These empirical findings partly and indirectly explain the insignificant effect of positive information in our study since the information provided in our experiment was targeted at the production and environmental effects of GM technology for edamame growers. These results also imply that marketers of GM edamame should consider varied types of information that more directly involve benefits to consumers such as nutritional benefits, health benefits, or society benefits to counter the effects of negative information.

Our results suggest that consumer resistance toward GM foods is quite strong in the market and hence the introduction of new GM edamame would not be easy. Our results also showed that consumers significantly increase the choice of non-GM edamame relative to GM edamame after being provided with negative information on GM. Interestingly, consumers generally preferred GM edamame to unlabeled edamame, and this preference did not change with additional information about GM. This suggested that consumers like to be informed about what they are buying and consuming. Hence a strategy of not conveying any information and waiting for negative information to surface would likely fail as well in marketing GM products.

Overall, it is not surprising that the negative information effects outweigh the positive information effects. Similar findings have been found in a number of previous studies [3, 4]. What is perhaps more interesting from our results is that the order of provision of information does not matter given the stronger effects of negative information. This finding has significant implications for GM food marketing and policy. For example, negative information about GM technology disseminated by anti-GM groups would be hard to overcome once it is heard and processed by consumers. Our results basically imply that it does not matter if pro-GM groups counter the negative information by positive information as consumers will still put more weight on the negative information and act accordingly in the market. Equally important is that it would not matter if the pro-GM groups provide their information before or after the announcement of the negative information from the anti-GM groups.

## Supporting information

S1 Appendix Experimental instructions and survey questionnaire. (DOCX)

S2 Appendix Question used to form attitude variable towards GM food. (DOCX)

S3 Appendix Raw data. (XLSX)
