# Supplementary material for: Information and order of information effects on consumers’ acceptance and valuation for genetically modified edamame soybean
Source: PLoS One. 2018 Oct 24;13(10):e0206300. doi: 10.1371/journal.pone.0206300 (PMC6200256; doi:10.1371/journal.pone.0206300)
Supplement: S1 Appendix — (DOCX) [file pone.0206300.s001.docx]

**Supporting Information**

**S1 Appendix. Experimental Instructions and Survey Questionnaire**

**Information Letter**

**Emailed to Food Science Database using Survey Monkey**

Dear potential participants,

The Sensory Science Laboratory (Department of Food Science) is conducting a research project on a food product.

The experiment will take 30 to 45 minutes. After completing both sensory testing and decision-making experiment, you will receive a Wal-Mart gift card ($25). For the decision-making experiment, you will be asked to bring some cash to buy a frozen food product for later in-home consumption. Please bring some cash and change (< $10).

Participation is voluntary. Even if you decide to participate, you may withdraw from the project at any time without giving a reason and without any academic penalty. Your decision to withdraw will have no negative consequences.

If you are interested in this study, please follow the link http://xxxxxx/xx/xx to register for a set of time slots for the sensory evalutaion and decision-making experiment. You will be selected on a first-come first-serve basis for a particiaption time on August 20 or 21. If you have questions, please contact me at (xxx@xxx.xxx).

Thank you for considering this invitation,

Elijah Wolfe

AEAB Research Assistant

**Recruitment Survey**

Please complete all questions on this form.

1. Gender: □ Male □ Female
2. Age: ______________ years old
3. Ethnic background:

| □ White / Caucasian | □ Asian |
| --- | --- |
| □ Black / African American | □ Native American |
| □ Hispanic / Latin American | □ Others (Specify: ____________) |

1. Food Allergy: Do you have any known allergies to foods or odors?

| □ No |
| --- |
| □ Yes (Specify:________________________). |

1. Please select products you would not be willing to consume (check all that apply).

| □ Tofu | □ Bell Peppers |
| --- | --- |
| □ Chocolate | □ Carrots |
| □ Edamame | □ Milk |

1. Are you unfamiliar with any of these products? Only select products that you have never heard of.

| □ Tofu | □ Bell Peppers |
| --- | --- |
| □ Chocolate | □ Carrots |
| □ Edamame | □ Milk |

1. Are you allergic to any of these products? Only select product you are allergic to.

| □ Tofu | □ Bell Peppers |
| --- | --- |
| □ Chocolate | □ Carrots |
| □ Edamame | □ Milk |

1. Would you be available for the following time slots? (*dates can be changed*)

| Thursday, August 20, 2015 | Friday, August 21, 2015 |
| --- | --- |
| □ 08:00 a.m. - 9:00 a.m. | □ 09:00 a.m. - 10:00 a.m. |
| □ 10:00 a.m. - 11:00 a.m. | □ 10:00 a.m. - 11:00 a.m. |
| □ 11:00 a.m. - 12:00 p.m. | □ 11:00 a.m. - 12:00 p.m. |
| □ 12:00 p.m. - 1:00 p.m. | □ 12:00 p.m. - 01:00 p.m.  □ 1:30 p.m. - 02:30 p.m. |

**Consent to Participate in a Research Study**

Principal Researcher: Elijah Wolfe

Faculty Advisor: Michael Popp

**INVITATION TO PARTICIPATE**

You are invited to participate in a research study about Edamame soybean. You are being asked to participate in this study because you have indicated no food allergies to Edamame or soy products

**WHAT YOU SHOULD KNOW ABOUT THE RESEARCH STUDY**

*Who is the Principal Researcher?*

Elijah Wolfe. Graduate Research Assistant. Department of Agricultural Economics and Agribusiness.

*Who is the Faculty Advisor?*

Michael Popp. Professor. Department of Agricultural Economics and Agribusiness.

*What is the purpose of this research study?*

The purpose of this study is to perform a sensory evaluation involving taste, smell, touch, sight and overall impression of Edamame soybean grown using different technology and determine associated willingness to pay for the Edamame products.

*Who will participate in this study?*

120 panelist will be pre-screened through Survey Monkey to: i) attend an Edamame sensory evaluation; ii) actually bid on Edamame; and iii) fill out a survey. Participants will be screened to gain access to participants that have previously eaten Edamame products. Participants are adults 18 years or older and will be selected on a first-come, first-serve basis.

*What am I being asked to do?*

Your participation will require the following:

- Sensory evaluation of three Edamame products.
- Cash auction bids on these three Edamame products (only randomly selected participants will actually pay for their bids).

*What are the possible risks or discomforts?*

Risk includes food allergies of the edible soybean food product Edamame. Participants will be asked to bid for Edamame, but can choose to bid zero if they do not want to pay for the Edamame. Participants have also been prescreened for Edamame allergies.

*What are the possible benefits of this study?*

Results of this study will be used to assess the market for Edamame products.

*How long will the study last?*

All sensory evaluations and auction experiments are scheduled for the period of Aug. 20 to 21, 2015 and will last approx. 30 minutes per group of participants.

*Will I receive compensation for my time and inconvenience if I choose to participate in this study?*

Using survey monkey the participants will chose a time slot of their choosing and receive a $25 Wal-Mart gift card at the completion of their sensory evaluation, survey completion and bidding process.

*Will I have to pay for anything?*

An experimental auction will take place during your participation. Bids for three Edamame products will occur in three rounds. One of the rounds and one of the products will be selected as the binding product. Of the 15 prices bid, one of them will be randomly selected by a random N^th^ number. The N^th^ bid will become the market price for the Edamame. All participants who bid more than the market price will pay the market price for the binding Edamame product. Not everyone is guaranteed to walk out with an Edamame product. Only bidders who bid more than the randomly drawn market price for the binding product. No price ceiling will be included; however, at any time, you may choose to bid a zero amount if you truly do not wish to purchase the product at any price, so you will not be required to pay for anything unless you want to. However, even if you bid a price and it is not high enough, you will not receive Edamame and also not have to pay.

*What are the options if I do not want to be in the study?*

If you do not wish to be in the study, you are free to leave.

*How will my confidentiality be protected?*

All information will be kept confidential to the extent allowed by applicable State and Federal law.  ID#’s of participants will be distributed at random at the onset of the experiment and records linking ID#’s to individual participants will not be kept except to record whether participants appeared for their assigned time slot.

*Will I know the results of the study?*

At the conclusion of the study you will have the right to request feedback about the results. You may contact the faculty advisor, Michael Popp.

*What do I do if I have questions about the research study?*

You have the right to contact the Principal Researcher or Faculty Advisor as listed below for any concerns that you may have.

Elijah Wolfe. Graduate Research Assistant. Department of Agricultural Economics and Agribusiness. xxx@xxx.xxx.

Michael Popp. Professor. Department of Agricultural Economics and Agribusiness. xxx@xxx.xxx.

You may also contact the University of Arkansas Research Compliance office listed below if you have questions about your rights as a participant, or to discuss any concerns about, or problems with the research.

Ro Windwalker, CIP

Institutional Review Board Coordinator, Research Compliance

E-mail: xxx@xxx.xxx

I have read the above statement and have been able to ask questions and express concerns, which have been satisfactorily responded to by the investigator. I understand the purpose of the study as well as the potential benefits and risks that are involved. I understand that participation is voluntary. I understand that significant new findings developed during this research will be shared with the participant. I understand that no rights have been waived by signing the consent form. I have been given a copy of the consent form.

Signed: ____________________________ Date:_____________________

**General Instructions**

You will be participating in an experiment about consumer decision making.

Please read and follow the instructions carefully.

**Specific Instructions**

You will receive a $25 Walmart gift card for participating in this experiment. It will be given to you at the end of the taste test and auction. This money is payment for the amount of time you devote to our session today. Please do not think of this as money that you can use to purchase the products that will be auctioned today. Risks of food allergies are present so please be cautious when making a personal decision to consume any of the products.

The first part of the experiment involves a simple sensory analysis. There will be three Edamame products to analyze using sight, smell, touch, and taste. There will be a few survey questions to answer after sampling the products.

The second part of the experiment involves an auction in two stages. Stage 1 is a practice round in which you will be asked to decide how much you would be willing to pay for candy bars. In stage 2 you will be asked to decide how much you would be willing to pay for the Edamame products you tasted.

You will submit your bids by writing them on bid sheets. You cannot reveal your bids to any other participant. After the auction, participants will fill out a short questionnaire.

**Please STOP here until the moderator tells you to continue.**

**Candy Bar Auction**

In a moment, we will give you the opportunity to participate in a practice, fake auction for candy bars. The purpose of this practice auction is to fully familiarize you with the auction procedures that we will use for the Edamame auction. Here in the front of the room are three candy bars with different ingredients.

We will give you the opportunity to practice the bidding process of the type of auction we will use later. Right now, you are bidding to obtain different types of candy bars. Here in the front of the room, we have Twix, Hershey Almond and Reese’s.

1. We will now conduct an auction, where you will have the opportunity to obtain a candy bar. In a moment, you will be asked to indicate the *highest* amount of money you are willing to pay *individually* for each of the three products by writing bids on the enclosed bid sheets. There will be two rounds of bidding. After the 2^nd^ round of bidding, a binding round and a binding product will be randomly chosen. Both of the rounds and each of the 3 products will have an equal chance of becoming the binding round and the binding product, respectively. A number (from 2 to the total number of subjects in your session) will also be randomly picked to determine the market price. The top N-1 bidders of the binding product in the binding round will then have to purchase the binding candy bar product using the N^th^ highest bid (the randomly picked N) as the price. So if for example, we randomly picked the number “4”, then the top 3 bidders of the binding product in the binding round will have to purchase the product at the market price, which is the 4^th^ highest bid. Note that if you are one of the winners, you will not have to purchase the product in this practice round or pay the corresponding market price. This IS a hypothetical exercise. If you are not interested in the product, you can bid a zero amount for the product.
2. Each of you has been given bid sheets in your packet. On this bid sheet you will, in a moment, write the ***highest*** amount of money you would be willing to pay for each product individually. Note: your bids are private information and should not be shared with anyone.
3. After you have finished writing your bids, the facilitator will go around the room and collect the bid sheets.

**Important Notes**

- You will only have the opportunity to obtain one of the products. Because we randomly draw a binding round and product, you cannot obtain more than one of the products from this auction.

- The winning bidders ***will not actually pay money*** to obtain the product that is binding. This procedure **is** hypothetical.
- In this auction, the best strategy is to bid ***exactly*** how much you are willing to *pay* to obtain each of the products individually.
- It is acceptable to bid $0.00 in any round. This would mean that you are not willing to pay for the product at any price.
- More importantly, we are interested in how much you would be willing to pay for the products.

Do you have any questions before we begin?

1. Now lets begin with the 1^st^ round. For this first round, please examine the 3 candy bar products. Now please indicate the maximum amount that you are willing to pay for each of the products in the bid sheets.
2. Now lets begin with the 2^nd^ round. “Almonds are good for your cardiovascular system!” Again, please indicate the maximum amount that you are willing to pay for each of the products in the bid sheets.
3. Now, we will randomly pick the binding round and randomly pick the binding product. We will then also randomly pick the “N” from 2 to the number of people in this session to determine the number of winners and the market price. The winners will be the top N-1 bidders of the binding product in the binding round. Each of these winners will then have to purchase the product at the market price, which is the N^th^ highest bid for the product.
4. The winners’ ID#s will be announced and will **not** have to pay the n^th^ highest bid amount for the candy bar product since this auction was fake and intended to teach you how to bid. Consequences of bidding too high or too low will be discussed after the auction is complete.

**Edamame Auction**

Now that you have had the chance to learn how the auction will work, we are interested in your preference for Edamame products.

We will give you the opportunity to participate in an auction to obtain different types of Edamame. Here in the front of the room, we have Non-GMO Edamame, unlabeled Edamame, and “genetically engineered” Edamame taken from soybean plots here at the Experimental research station where different varieties of soybean are grown.

1. We will now conduct an auction, where you will have the opportunity to obtain an Edamame product. In a moment, you will be asked to indicate the *highest* amount of money you are willing to pay *individually* for each of the three products by writing bids on the enclosed bid sheets. The procedures for this auction are similar to the candy bar auction. However, there will be three rounds of bidding. After all the 3 rounds of bidding, a binding round and a binding product will be randomly chosen. That is, we will randomly select the round and the product that will be the basis of your bidding activity today. Each of the 3 rounds and each of the 3 products will have an equal chance of becoming the binding round and the binding product, respectively. A number (from 2 to the total number of subjects in your session) will also be randomly picked to determine the market price. The top N-1 bidders of the binding product in the binding round will then have to purchase the binding Edamame product using the N^th^ highest bid (the randomly picked N) as the price. So if for example, we randomly picked the number “4”, then the top 3 bidders of the binding product in the binding round will have to purchase the product at the market price, which is the 4^th^ highest bid. Note that if you are one of the winners, you will have to purchase the product and pay the corresponding market price. This is no longer fake. Since we are choosing the binding product, you can only take home one product, not 2 or 3, if you are one of the winners. If you are not interested in the product, you can bid a zero amount for the product.
2. Each of you has been given bid sheets in your packet. On this bid sheet you will, in a moment, write the ***highest*** amount of money you would be willing to pay for each product individually. Note: your bids are private information and should not be shared with anyone.
3. After you have finished writing your bids, the facilitator will go around the room and collect the bid sheets.

**Important Notes**

- You will only have the opportunity to obtain one of the products. Because we randomly draw a binding round and product, you cannot obtain more than one of the products from this auction.

- The winning bidders ***will actually pay money*** to obtain the product that is binding.
- In this auction, the best strategy is to bid ***exactly*** how much you are willing to *pay* to obtain each of the products individually.
- It is acceptable to bid $0.00 in any round. This would mean that you are not willing to pay for the product at any price.
- More importantly, we are interested in how much you would be willing to pay for the products.

Do you have any questions before we begin?

1. Now lets begin with the 1^st^ round. For this first round, please examine the 3 Edamame products that you have just tasted during the sensory test. Now please indicate the maximum amount that you are willing to pay for each of the products in the bid sheets.
2. Now lets begin with the 2^nd^ round. Please carefully read this information sheet we are providing you before bidding on the products. After you have read this information sheet, please indicate the maximum amount that you are willing to pay for each of the products in the bid sheets.
3. Now lets begin with the 3^rd^ round. Please carefully read this information sheet we are providing you before bidding on the products. After you have read this information sheet, please indicate the maximum amount that you are willing to pay for each of the products in the bid sheets.
4. Now, we will randomly pick the binding round and randomly pick the binding product. We will then also randomly pick the “N” from 2 to number of people in this session to determine the number of winners and the market price. The winners will be the top N-1 bidders of the binding product in the binding round. Each of these winners will then have to purchase the product at the market price, which is the N^th^ highest bid for the product.
5. The winners will come forward, take the Edamame product, and pay the n^th^ highest bid amount for the Edamame product.
6. Winners will be given a certificate, reflecting any transactions agreed to, and distributed their Edamame.

### Questionnaire

All of your responses will be kept confidential. Please circle the number to the left of the answer, if one is provided.

**Edamame Consumption**

Please circle the appropriate number (choose one) next to the response below:

A1. How often did you eat edamame in the **PAST THREE MONTHS**?

0. Never

1. 1-5 times

2. 6-10 times

3. 11-15 times

4. 16 times or more

A2. How often did you buy edamame to prepare meals for your household in the **PAST THREE MONTHS** (e.g. grocery store, farmer’s market)?

0. Never

1. 1-5 times

2. 6-10 times

3. 11-15 times

4. 16 times or more

A3. How many servings of edamame did you buy away-from-home for your household in the **PAST THREE MONTHS** (e.g. restaurant)?

0. Never

1. 1-5 times

2. 6-10 times

3. 11-15 times

4. 16 times or more

**Opinions about Genetically Engineered Food**

Please circle the appropriate number (choose one) next to the response below:

B1. Regarding **genetically engineered food production technology used on farms**, how informed do you consider yourself?

0. Extremely well-informed

1. Well-informed

2. Somewhat informed

3. Not very informed

4. Not informed at all

B2. Regarding **genetically engineered foods**, how informed do you consider yourself?

0. Extremely well-informed

1. Well-informed

2. Somewhat informed

3. Not very informed

4. Not informed at all

B3. Planting RoundUp Ready^®^ soybean allows farms to grow soybean and spray RoundUp^®^ herbicide to control weeds without killing soybean whereas using RoundUp^®^ herbicide on conventional (non-genetically engineered soybean) would not only kill weeds but also the conventional soybean.

0. True 1. False 2. Not sure

B4. Some soybean oil sold in the U.S. is derived from Roundup Ready^®^ soybean.

0. True 1. False 2. Not sure

B5. In addition to Roundup Ready^®^ soybean, other genetically engineered crops are currently grown in the U.S.

0. True 1. False 2. Not sure

B6. Chemicals in RoundUp^®^ herbicide remain effective for weed control in the soil forever.

0. True 1. False 2. Not sure

B7. Do you think you have eaten genetically engineered food in the past month? (don’t count today’s study)

1. Yes

2. No

B8. How much would you say you’ve heard or read about genetically engineered foods?

1. Nothing at all

2. Not much

3. Some

4. A great deal

B9. How often have you discussed genetically engineered foods?

1. Frequently (typically once or more often per week over the last year)

2. Occasionally (no more than once a month in the last year)

3. Only once or twice over the last year

4. Never

| Question | Strongly Agree | Somewhat Agree | Somewhat Disagree | Strongly Disagree |
| --- | --- | --- | --- | --- |
| B10. Genetically engineered food such as Roundup Ready^®^ Soybeans present no danger for future generations. |  |  |  |  |
| B11. I think it is safe for me to eat genetically engineered food. |  |  |  |  |
| B12. Physical harm to mankind is bound to happen as a result of genetically engineered foods. |  |  |  |  |
| B13. Growing genetically engineered crops will be harmful to the environment. |  |  |  |  |
| B14. There are benefits to developing genetically engineered foods such as higher yields and a more sustainable food source. |  |  |  |  |
| B15. Small-scale farmers are negatively impacted by the development of genetically engineered foods as the cost of seed will be higher. |  |  |  |  |

B16. Who would you trust the most to provide you information about genetically engineered crops (Please circle **THREE**)?

1. Research institutions

2. Seed Technology Companies

3. FDA

4. Universities

5. Religious Groups

6. Media (Fox, CNN, etc)

7. Farmer groups

8. Non-GMO Project Groups

9. Social Media (Facebook, etc)

10. USDA

12. Friends and Family

11. Others (please specify) __________________________________

**Your Information**

C1. What is your gender?

0. Male 1. Female

C2. How old are you?

____ years old

C3. Are you a current University of Arkansas student?

0. No

1. Yes

C4. What is the highest education level you have completed?

0. Less than high school

1. High school/GED

2. Some college/2 year associate degree

3. Bachelor’s degree

4. Master’s degree

5. PhD

6. Other ______________

C5. What is your current employment status?

1. Employed part time (fewer than 40 hours per week)

2. Employed full time (40 or more hours per week)

3. Retired

4. Other (please specify): __________

**Your Household Information**

Depending on your situation, we would like you to think of your household as the person or persons who you are financially responsible for, including yourself, even if you are not a wage earner.

C6. How many people, including yourself, are in the following age categories in your household?

Age 5 and younger ____

Age 6 to 17 ____

Age 18 to 39 ____

Age 40 to 54 ____

Age 55 and above ____

C7. How much money do you typically spend on groceries per week for your household?

0. None

1. $1-$50 per week

2. $51-$100 per week

3. $101-$200 per week

4. $201 or more per week

C8. How much do you typically spend on away-from-home food per week for your household?

0. None

1. $1-$50 per week

2. $51-$100 per week

3. $101-$200 per week

4. $201 or more per week

C9. What is your total **household monthly allowance and/or income before taxes** from all sources, including family or other sources of economic support that is used to support your household)?

| 0. | Less than $999 | 5. | $5,000 - $5,999 |
| --- | --- | --- | --- |
| 1. | $1,000 - $1,999 | 6. | $6,000 - $6,999 |
| 2. | $2,000 - $2,999 | 7. | $7,000 - $7,999 |
| 3. | $3,000 - $3,999 | 8. | More than $8,000 |
| 4. | $4,000 - $4,999 |  |  |

C10. What percentage (%) of your total grocery purchase dollars are spent at the following stores?

____ Wal-Mart Supercenter

____ Harps/Price Cutter

____ Ozark Natural Foods

____ Sam’s Club

____ ALDI

____ Marvin’s Savers Club

____ Wal-Mart Neighborhood Market

____ Farmer’s Markets

____ List Others ____________

**100%**

C11. Please select how important each factor is to you in making a grocery purchase.

| Factors | Not at all Important | Slightly  Important | Moderately  Important | Very Important | Extremely Important |
| --- | --- | --- | --- | --- | --- |
| Appearance |  |  |  |  |  |
| Brand |  |  |  |  |  |
| Price |  |  |  |  |  |
| Expiration Date |  |  |  |  |  |
| Organic production |  |  |  |  |  |
| Non GMO production |  |  |  |  |  |
| Eco packaging/Recyclable |  |  |  |  |  |
| Package Size |  |  |  |  |  |
| Locally produced |  |  |  |  |  |
