# Supplementary material for: Information and order of information effects on consumers’ acceptance and valuation for genetically modified edamame soybean
Source: PLoS One. 2018 Oct 24;13(10):e0206300. doi: 10.1371/journal.pone.0206300 (PMC6200256; doi:10.1371/journal.pone.0206300)
Supplement: S2 Appendix — (DOCX) [file pone.0206300.s002.docx]

**Supporting Information**

**S2 Appendix.** **Question Used to Form Attitude Variable towards GM Food**

| Question | Strongly Agree | Somewhat Agree | Somewhat Disagree | Strongly Disagree |
| --- | --- | --- | --- | --- |
| Genetically engineered food such as Roundup Ready^®^ Soybeans present no danger for future generations. | □_1_ | □_2_ | _3_ | □_4_ |
| I think it is safe for me to eat genetically engineered food. | □_1_ | □_2_ | _3_ | □_4_ |
| Physical harm to mankind is bound to happen as a result of genetically engineered foods. | □_4_ | _3_ | □_2_ | □_1_ |
| Growing genetically engineered crops will be harmful to the environment. | □_4_ | _3_ | □_2_ | □_1_ |
| There are benefits to developing genetically engineered foods such as higher yields and a more sustainable food source. | □_1_ | _2_ | □_3_ | □_4_ |
| Small-scale farmers are negatively impacted by the development of genetically engineered foods as the cost of seed will be higher. | □_4_ | □_3_ | □_2_ | _1_ |

Note: The attitude score is the average of values assigned to each of the agreement levels for each statement. The score represents a summary of all rankings of statements made. Note that some statements are reverse scored to reflect a consistent estimate of the degree of concern over genetically engineered food. Participants with an average opinion score of 1 are in favor of genetically engineered food whereas a score of 4 reveals the opposite. The above example averages to 2.5 indicating a neutral sentiment toward genetically modified food although each statement may well carry different weight from individual to individual and unitary difference across levels of agreement are again an approximation.
